# Targeting Telomeres and Telomerase: Studies in Aging and Disease Utilizing CRISPR/Cas9 Technology

**DOI:** 10.3390/cells8020186

**Published:** 2019-02-21

**Authors:** Andrew C. Brane, Trygve O. Tollefsbol

**Affiliations:** 1 Department of Biology, University of Alabama at Birmingham, 1300 University Boulevard, Birmingham, AL 35294, USA; brane@uab.edu; 2 Comprehensive Center for Healthy Aging, University of Alabama Birmingham, 1530 3rd Avenue South, Birmingham, AL 35294, USA; 3 Comprehensive Cancer Center, University of Alabama Birmingham, 1802 6th Avenue South, Birmingham, AL 35294, USA; 4 Nutrition Obesity Research Center, University of Alabama Birmingham, 1675 University Boulevard, Birmingham, AL 35294, USA; 5 Comprehensive Diabetes Center, University of Alabama Birmingham, 1825 University Boulevard, Birmingham, AL 35294, USA

**Keywords:** telomeres, telomerase, CRISPR, CRISPR/Cas9, Cas9, dCas9, cancer, aging

## Abstract

Telomeres and telomerase provide a unique and important avenue of study in improving both life expectancy and quality of life due to their close association with aging and disease. While major advances in our understanding of these two biological mediators have characterized the last two decades, previous studies have been limited by the inability to affect change in real time within living cells. The last three years, however, have witnessed a huge step forward to overcome this limitation. The advent of the clustered regularly interspaced short palindromic repeats/CRISPR-associated (CRISPR/Cas) system has led to a wide array of targeted genetic studies that are already being employed to modify telomeres and telomerase, as well as the genes that affect them. In this review, we analyze studies utilizing the technology to target and modify telomeres, telomerase, and their closely associated genes. We also discuss how these studies can provide insight into the biology and mechanisms that underlie aging, cancer, and other diseases.

## 1. Introduction

### 1.1. Telomeres and Telomerase

Since their discovery, telomeres have been at the forefront of research in both aging and disease. There were observations implicating structures on chromosome ends assisting in stability as far back as the 1930’s, but it was not until Blackburn and Gall’s pioneering paper that the modern idea of a telomere emerged [1,2]. Their initial study showed that telomeres consist of linked, repeating nucleotide hexamers. Further study revealed a variety of possible sequences among clades of organisms [3]. Within these clades, however, function is highly conserved, with telomeres being transferred from distantly related species that are able to maintain biological activity [4].

This activity is important for mitigating damage to genes during chromosomal replication. DNA polymerase is unable to replicate the ends of chromosomes, due to the nature of DNA replication. Telomeres act as buffer zones, which prevent the gradual degradation of genes. Over the lifetime of a cell, telomeres become shorter, and cells will become senescent once a critical length is reached [5]. Because it is necessary to restore telomeres to continue division and reproduction, the discovery of telomeres posed a mechanistic question as to how they are built and maintained. The answer to this question followed shortly after, when Greider and Blackburn discovered and isolated the protein that they called terminal transferase [6]. This protein is today known as telomerase. 

As a ribonucleoprotein, telomerase is composed of both RNA and proteins, and it consists of two moecules each of telomerase reverse transcriptase, telomere RNA, and dyskerin [7,8,9]. While each subunit is necessary for proper biological function, the catalytic portion that is known as telomerase reverse transcriptase (TERT and hTERT in humans) is normally the limiting factor for telomerase activity and telomere elongation [10]. It is for this reason that a majority of research regarding telomere biology focuses on TERT.

Because of their intimate association with cell replication and senescence, telomeres and, by extension, telomerase, have been implicated in disease and aging since the 1980’s [5]. Mice that were bred to be deficient in telomerase showed a marked decrease in health and, after several generations, lose the ability to breed completely [11]. Studies have shown that telomere length decreases in older organisms and that this effect is not simple correlation [12,13]. Within tissues, the ablation of telomeres results in spatially specific, age-associated damage [13,14]. On a cellular level, the loss of telomere function is linked with decreased ability for cellular division, and TERT overexpression is linked with an increase in cellular proliferation [15]. The effect is especially pronounced in stem cells, where telomerase is normally upregulated to a high degree [16,17]. When telomerase activity is disrupted in these cells, they lose replicative capacity and lose their pluripotency. In addition, this disruption leads to an increase in cellular oxidative stress [18].

**Figure 1:**
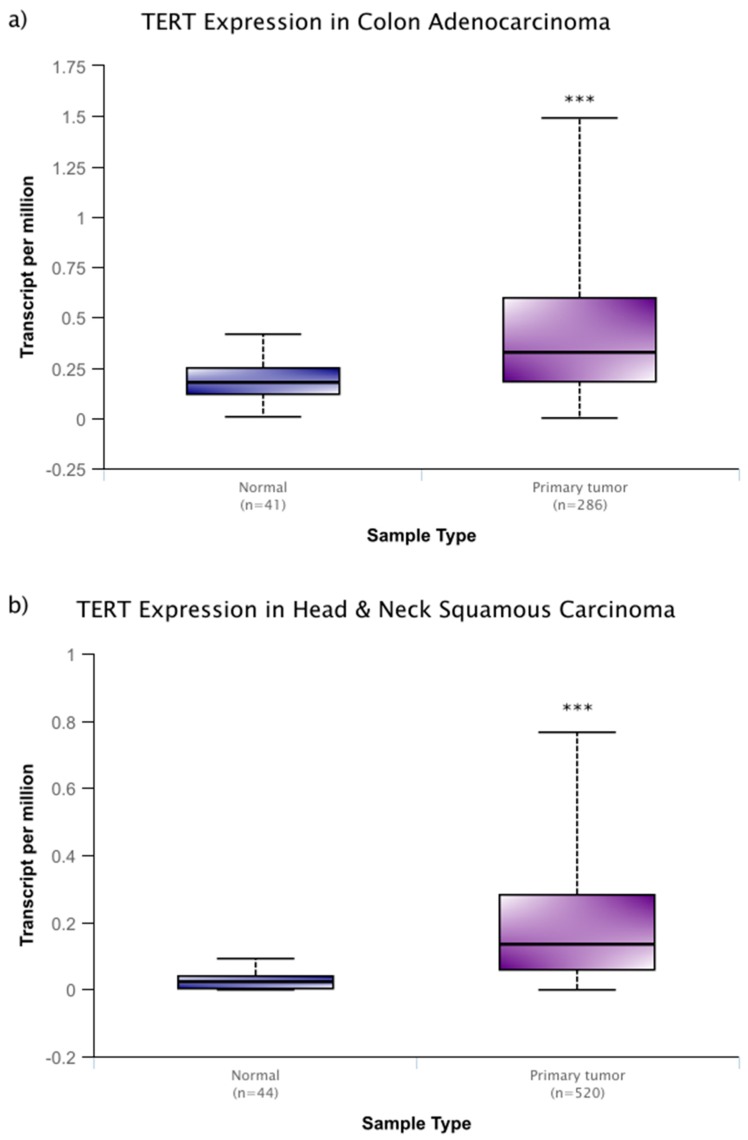

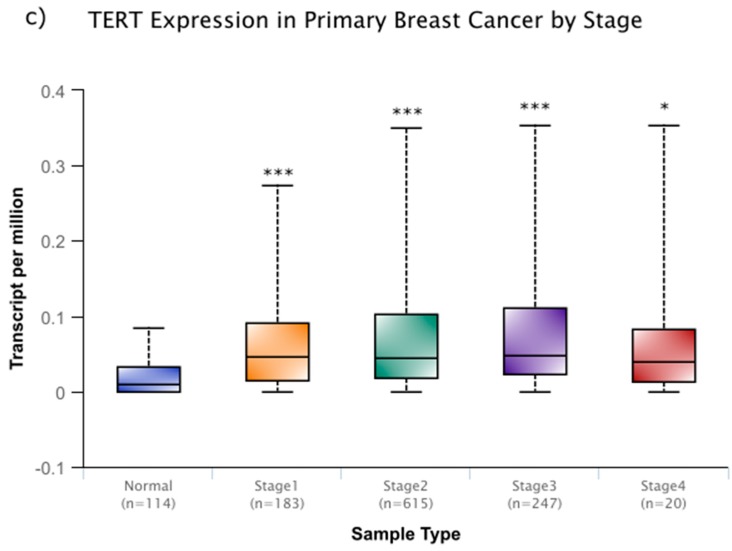
Telomerase reverse transcriptase (*TERT)* expression of colon adenocarcinoma (**a**), and head and neck squamous carcinoma (**b**), and primary breast cancer (**c**) within the publicly available National Cancer Institute Genomic Data Commons. In (**c**), *TERT* expression data is expanded based on stage of breast cancer sampled. While there is no significant difference among cancer stages, each stage of cancer displays significantly higher expression than normal tissue. Data were accessed through the University of Alabama at Birmingham UALCAN cancer transcriptome database (http://ualcan.path.uab.edu) [20]. These box and whisper plots encompass all transcriptome data recorded, with the upper and lower bars giving the total range of data recorded. The bolded middle line represents the mean of the data, and the second and third quartiles are contained within the box. Significance in relation to normal tissue is denoted by asterisks, with * representing a *p* < 0.05 and *** representing a *p* < 0.001. These types of cancer (**a**–**c**) were chosen for their relatively high sample sizes, but these trends carry across many other cancer types.

However, these changes in activity are not solely linked with aging. As is the case with many cellular processes, disruptions to the normal function of telomeres and telomerase are associated with human disease. One of the most closely associated of these diseases is, perhaps, cancer. In around 90% of cancers, the expression of telomerase is increased, while similar, yet benign, tumors do not display this increase in telomerase [19]. In a sample that was derived from cancer patient data within the National Cancer Institute Genomic Data Commons, this increase is seen across several cancer types (Figure 1a–c) and it remains high through progressive breast cancer stages (Figure 1c) [20]. In addition, more severe, metastatic stages of cancer experience higher expression levels of telomerase, and disrupting telomerase activity may have some efficacy in preventing metastasis [10,21]. It is thought that these high levels of expression prevent cellular senescence and they allow for aggressive, rapidly dividing cancer lines.

### 1.2. The CRISPR-Cas System

While major strides in the understanding of the function and dysfunction of telomeres and telomerase have been apparent, a crucial hurdle in this study has been the inability to affect and observe changes within living systems. However, recently a solution to this issue has emerged. The clustered regularly interspaced short palindromic repeats-CRISPR-associated (CRISPR-Cas) system was first described as an adaptive bacterial immune system in the early 2000’s and it functions to attack foreign bacterial and viral DNA in prokaryotes [22]. This discovery, named for its genomic clustered regularly interspaced short palindromic repeats (CRISPR) and CRISPR-associated (Cas) protein, drew interest from an evolutionary standpoint at the time. It was not until eight years later that an application for this system would be discovered and would draw wider acclaim. Between 2013 and 2014, the laboratories of Drs. Feng Zhang and George Church described methods of editing DNA in vitro utilizing the CRISPR-Cas system [23,24]. It was not long after that many other investigators began to adopt and modify the system.

This system is comprised of two major parts, which can be roughly broken down into a guiding and an affecting portion. Responsible for specificity, the guiding portion consists of a single stranded RNA molecule, which is called single guide RNA (sgRNA) [25]. This RNA component targets a genomic region by complementing a specific DNA sequence and is associated with the affecting portion by way of a fused portion of scaffold RNA. This affecting component is comprised of one of several Cas proteins, the most common being Cas9. In its native state, this protein has double-stranded endonuclease activity [24,26]. After producing a cut in the DNA, donor DNA with a desired sequence can be added to the target site [27]. Combining this endonuclease activity with the aforementioned RNA guide allows for highly specific, tightly regulated editing of genetic information in vitro and in vivo.

**Figure 2:**
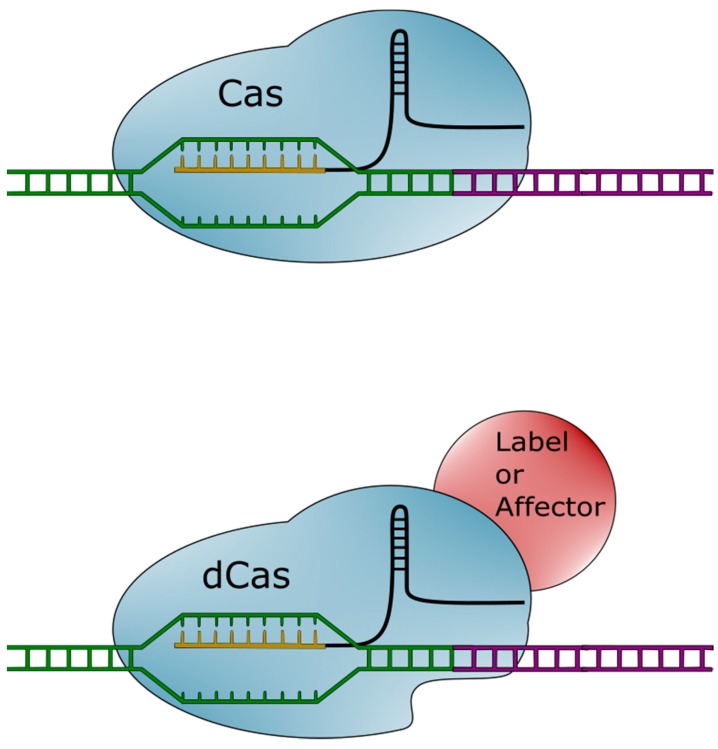
Diagram of clustered regularly interspaced short palindromic repeats-associated (Cas) system variants. The Cas (upper) system targets a specific genomic region (green) with its single guide RNA (sgRNA) (gold and black). The Cas protein will then make a cut in the adjacent DNA region (purple). The catalytically inactive Cas (dCas) (lower) system targets a genomic region with an identical system. However, the dCas protein lacks endonuclease activity. Various molecules (red) can be fused to the dCas protein. These include labels and affectors. Labels bring a fluorescent signal in close proximity to target DNA, while affectors can modify characteristics, such as the epigenetic state of DNA.

The CRISPR-Cas system is not, however, limited to inducing double-stranded breaks. The Cas protein can be modified to retain its targeting ability while losing its endonuclease activity [25]. This catalytically inactive Cas (dCas) can be used as is, or further modified with a number of different functional groups. Molecules (visualized in Figure 2) can be attached to dCas. These molecules can then be brought into proximity or attached to specified regions of the genome.

## 2. Telomeres—Imaging

One emerging use of this CRISPR-dCas system involves targeting telomeres for imaging. This system has a number of advantages over other systems, most of which stem from the dynamic and sustained nature of the CRISPR system. In one of the first imaging experiments involving CRISPR, Chen et al. were able to label telomeres in HEK293T, UMUC3, and HeLa cell lines with enhanced green fluorescent protein (EGFP) [28]. Within these cells, telomere movements were observed with a labeling efficiency and intensity akin to the well-established DNA FISH protocol. Further improvements that were made on this system may, in fact, result in greater labeling efficiency and specificity [29]. By replacing the EGFP with the brighter mClover fluorescent tag, labeled telomeres became even easier to detect and only produced negligible off-target effects [30]. Imaging telomeres is not in itself a new idea, but the unprecedented precision and efficiency of the CRISPR-Cas system provides a novel way to quickly and efficiently track telomeres.

The true advantage of CRISPR imaging is, however, its ability to be directly applied to living systems. While there are other methods of fluorescently labeling genomic elements, these are toxic to the cell and can result in irreparable DNA damage [31]. This limitation has disallowed the uninterrupted recording of telomeres and other genomic elements in vitro. Shao et al. were among the first to establish that labeling with the CRISPR-dCas had minimal cytotoxicity and was suitable for continuous viewing [32]. Their system was used to track telomeres and centromeres over a five-minute period and measured the relative movements of each during interphase.

Building on these ideas, Dreissig et al. were able to track telomere movements in leaf cells of *Nicotiana benthamiana* using dCas that was labeled with both EGFP and mRuby2 fluorescent tags [33]. Within the nucleus, they observed telomere movements of up to 2 μm during interphase. In addition, combining this technique with fluorescently labeled proteins allowed for the visualization of live protein-telomere interactions. By labeling both telomeres and end-binding protein TRB1, they found that these leaf cells appeared to contain chromosomes with both blunt and overhanging ends, a phenomenon that is not observed in mammals or fungi. While this study was limited to this single, specific protein interaction, future work could lead to an understanding of how telomeres interact with any relevant portions of the proteome.

More recently, this technique has been extended to transgenic mouse models [34]. By expressing dCas-GFP throughout a mouse, the guides for telomeres could be inserted into specific tissues for labeling. The group used this technology, combined with CRISPR-interference of the TRF1 gene, to observe the aggregation and fusion of telomeres in real time. This technology has the potential to be extended to other genes, allowing for the study of real-time changes in telomere dynamics after genetic manipulations.

## 3. Telomeres—Editing

As discussed earlier, disruptions and damage to telomeres can lead to a wide array of cellular dysfunction. The CRISPR-Cas system’s ability to cut and insert genes allows for the real-time, in vivo study of telomere damage. Using this system to induce double strand breaks (DSBs) in telomeres resulted in the activation of a telomeric repair system that was regulated by the Rad51 gene [35]. This study differs from previous conflicting and ambiguous results, which are likely due to a lack of precision in non-CRISPR induction of DSBs. Previous findings were clouded by the initiation of senescence and apoptotic pathways in DSB-induced cells, and this study provides an example of how the Cas system can be utilized to remove the noise from results.

Taking these ideas a step further, Kim et al. were able to completely remove telomeres in bone marrow neuroblasts and measure the effects on cellular function and senescence [36]. Telomere removal led to a cascade of cellular changes, chiefly a loss of mitochondrial function and an aggregation of Parkinson’s disease (PD) associated proteins. This change lowered cellular viability and it has the potential to model both aging and PD in cells. Because this method only removes telomeres, it allows for the study of how this specific process contributes to cellular aging [13,14,36]. This process is important in establishing causality and removing ambiguity that could be associated with other cellular aging models. 

CRISPR-Cas can also be employed to create more minor changes to telomeres. While the changes can be as small as a single nucleotide, they can have a major impact on a cell’s biology. After inducing a mutation to a subtelomeric CTCF binding site, known as TERRA, the cells exhibited a loss of sister telomeres and reduced capacity for replication [37]. These issues were exacerbated by the induction of replication stress and it led to a higher rate of apoptosis. This study implicates CTCF and TERRA sites as being vital for successful telomere replication and maintenance and elucidates their importance for the overall maintenance and stability of chromosomes.

While current research involving CRISPR-Cas ablation of telomeres has been limited, the ability to use the technology in any cell type allows for the study of a variety of different diseases. By utilizing these methods in different tissues, the effect of aging can be measured across a broad array of conditions. Further study has the potential to answer both biological and mechanistic questions regarding telomere loss and the disease states that it causes.

## 4. Telomerase—Imaging

While imaging the genomic region containing *TERT* with dCas is possible, it is not the nucleotide sequence itself that is primarily associated with biological function. Because of this, targeting and modifying the protein telomerase appears to be the most effective way to image and study its dynamics. By introducing a fluorescent marker at the *TERT* locus, Schmidt, Zaug, and Cech were able to distinguish three stages of telomerase movement [38]. The stages can be characterized as a rapid diffusion stage, a frequent, transient telomere-associating stage, and a rarer, long-term association stage that results in a majority of telomere elongation. In addition, telomerase appears to bind with the ssDNA overhangs and add multiple hexamer repeats in tandem [39]. Taking these results together provides a novel model for telomere formation; wherein, telomeres are elongated in short controlled periods following longer periods of transient association. Labeling and the subsequent imaging of telomerase using the CRISPR-Cas system allows for an unprecedented ability to study the spatiotemporal dynamics of telomerase movements and recruitment. Understanding these dynamics is vital in the study of diseases, such as cancer, which utilize the protein to facilitate rapid, aggressive division.

## 5. Telomerase—Editing

One of the most common and valuable tools in biotechnology is modulating gene expression by knocking out or knocking in a gene. When doing so, comparing differences to wild type organisms allows for the parsing of genetic function. By targeting the promoter of *hTERT*, CRISPR-Cas can be used to both ablate and enhance TERT expression [40]. Mutations that led to silencing resulted in normally immortal cell lines senescing and eventually dying, while those that increased expression saw TERT levels that were akin to those found in tumor cell lines. As an unintended consequence of adding a protein Halo tag to the *N*-terminus of TERT, Chiba et al. found that they could modulate expression between these two extremes [41]. Additionally, they found a reduction of telomere lengths within these cell lines, implicating steric hindrance as a factor in telomere lengthening.

A different study by Xi et al. explored similar ideas, focusing on urothelial cancer cells [42]. These cells contain a single DNA substitution mutation in their *hTERT* promoter, which had previously been associated with high expression levels. The group used the CRISPR-Cas system to revert this mutation and observed a restoration in the baseline *hTERT* levels. 

In a later study, the mechanism behind this phenomenon was explored. In this experiment, promoter mutations were induced by the CRISPR-Cas system [43]. These mutants saw an increase in chromatin interactions upstream of the gene, as well as a recruitment of the transcription factor GABPA. This transcription factor directly recruits DNA polymerase II and it provides a possible mechanism for the activation of *TERT* caused by promoter mutations across multiple cancer types.

While targeting telomerase directly has important clinical implications in cancer treatment, there are still 10–15% of cancer cases that exhibit telomere lengthening without a corresponding increase in telomerase activity [19]. These remaining cancers are still able to replicate rapidly, so they must be lengthening their telomeres and preventing senescence by some other means. This alternative means of telomere lengthening (ALT) is the proposed mechanism for this prevention and it must be studied in order to achieve a full understanding of cancer proliferation [44]. One way to increase understanding of these ALT pathways is to generate a continuously dividing cell line that lacks telomerase activities. By utilizing CRISPR to knockdown both TERT and a cell death pathway that is known as ATRX/DAXX, cells that exhibited ALT pathways were formed. This ALT pathway can also be achieved through CRISPR mediated knockout of the RNA component of telomerase (*TERC*) [45]. The ALT pathway arose in only a tiny fraction of cells and led to telomere generation with large overhangs on the lagging strands. While ALT telomere elongation appears to be rare and it is still not fully understood, unraveling the mechanism and biology of the process is important for studying cancer that does not employ telomerase-associated telomere lengthening.

Due to their prevalence in the disease, understanding the significance of TERT mutations is vital in the understanding of cancer cell growth. CRISPR-Cas provides a powerful tool for affecting these mutations in live cells and it allows for the modeling of rapidly dividing cell lines. However, this modeling is not limited to cancer research. It has great potential to model aging as well as diseases that are characterized by cellular aging. The technique has already been used to reprogram hTERT in fibroblast cells, creating a novel model for Werner’s syndrome [46]. With the ever-increasing understanding of disease mechanisms, it is even more important to be able to create accurate cellular models for these diseases. These models serve as a platform to test new drugs and therapies. It is therefore important that they have accurate, specific genetic states to ensure that treatments will translate from the laboratory into patients. 

## 6. Genes that Affect Telomeres and Telomerase

As with any gene or protein within a biological system, telomeres and telomerase are affected by a suite of different genes. The CRISPR-Cas system allows for the identification and subsequent modification of these genes. Modifications to these genes often induce changes that are associated with cancer and aging, but some disease models can be induced independently of the two. One such gene is nuclear assembly factor 1 (NAF1) [47]. Within the cellular and mouse models CRISPR induced mutations of the NAF1 gene result in a loss of around half of cellular TERT activity. This mutation and the resulting expression loss form a profile that matches that of pulmonary fibrosis-emphysema. It is likely that this disease progresses by disrupting telomere homeostasis, a process that many aging and cancer-associated genes also influence.

In one of the earlier experiments to utilize the technology, CRISPR-Cas was used to confirm that the cold inducible RNA-binding protein (CIRP) functions in telomere maintenance at all temperatures and it modulates *TERT* expression at low temperatures [48]. With this gene knocked out, the overall telomere length was shorter than the controls. These results indicate that CIRP is necessary for mediating telomerase activity during hypothermia as well as under normal cellular conditions. 

Likewise, *Notch1*, a gene that is normally involved in development, was also found to be necessary for proper telomerase function [49]. Without it, telomeres shortened and expressed phenotypes that are typical of aging cells. This result also exemplifies Notch1’s role in cancer, due to evidence that telomeric shortening is important for early tumorigenesis [12,49]. The loss of telomeres leads to chromosomal instability, which is conducive to the development of cancer phenotypes. This change may also explain Notch1’s pleiotropic association with tumor suppression and oncogenesis, as the shortening of telomeres is also associated with decreased replicative capacity [50].

Gu et al. saw similar results with a different gene and mechanism; the group found that, by disrupting CTC1, a part of the telomere-regulating complex, telomeres would undergo rapid elongation, followed by an acute breakdown [51]. Similar to Notch1, this breakdown leads to chromosomal instability. Another gene that was implicated as important for genomic stability was *POLD3* [52]. Without it, cells lost telomeres, and these losses were likely due to the induction of DSBs. Cells that were deficient in POLD3 were unable to replicate efficiently and tended to have micronuclei. Ablating genes, such as these with CRISPR/Cas, allows for the systematic study of the mechanisms of telomere and telomerase loss.

In some cancer types, telomere fusions result in massive rearrangements of genes on chromosomes as well as on localized hypermutation [53]. By using the CRISPR knockdown array, the group found evidence that these genomic events are caused by recombination after activity from the cytoplasmic nuclease TREX1. As both rearrangements and hypermutation can lead to complications in recognizing and treating cancer cells, understanding how they are formed as well as what genetic factors contribute to them further the understanding of the disease itself. The identification of TREX1 provides an important screening target for clinicians when considering treatment and genetic counseling. 

## 7. Epigenetics—Editing

A critically important, yet understudied, avenue of telomerase biology is epigenetics. As epigenetic modifications can affect expression to a large degree, genes that modulate this expression are important targets for study. While there are no CRISPR-based studies involving the direct methylation or demethylation of the *hTERT* gene, there is some interest in the genes that enact these epigenetic changes. Cells that were deprived of the DNA methyltransferase 2 (DNMT2), which catalyzes the addition of methyl groups to tRNA, suffered both a decrease in telomere lengths and telomerase activity [54,55]. Interestingly, the loss of DNMT2 resulted in the compensatory upregulation of other DNA methyltransferases, including Dnmt1, Dnmt3a, and Dnmt3b. These methyltransferases primarily methylate DNA, which led to global DNA hypermethylation. In turn, this hypermethylation induced cellular senescence apoptotic pathways. These results suggest that DNMT2 could serve as an important target for cancer and other telomere-associated disease.

Conversely, Cas-mediated knockout of the ten eleven translocation (Tet) proteins, which facilitate DNA demethylation, resulted in an elongation of telomeres [56]. Although the loss of a demethylator would suggest a higher level of methylation and results that are in line with those of the DNMT2 study, the lack of compensatory global methylation likely prevented telomere shortening and cell senescence [55,56]. This result directly implicates Tet in the maintenance of normal telomere lengths and underscores its importance as a target for cancer and aging therapies. Understanding how changes in methylation state are induced at the *TERT* locus is vital, as they may provide insight as to why the expression levels differ in disease states with no obvious mutation.

## 8. Conclusions

**Figure 3:**
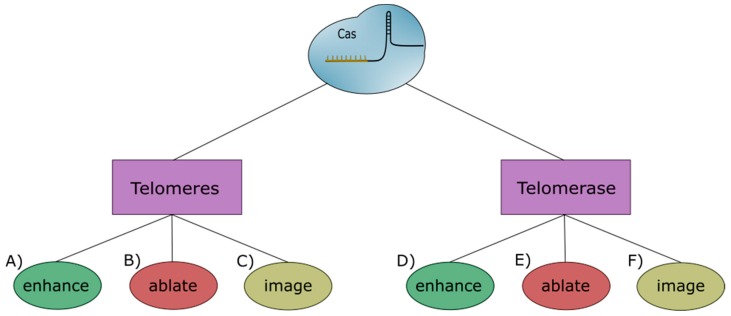
Overview of the uses for clustered regularly interspaced short palindromic repeats (CRISPR) in regards to telomeres and telomerase. Studies measuring an increase in telomere length (A) achieve this by activating telomere repair systems or increasing gene expression of proteins that build directly onto telomeres. As a whole, these studies observe normal to enhanced replicative capacity. Conversely, CRISPR-mediated ablation of telomeres occurs through direct removal and damage (B). These studies witness an upregulation of repair mechanisms as well as decreases in both cellular health and viability. Studies involved in imaging telomeres largely utilize fluorescently labeled dCas (C) and observe minimal cytotoxicity and high efficiency. These properties allow for real-time, in vivo study of telomere movements and interactions. Enhancing telomerase activity through interactions with other proteins and epigenetic changes (D) leads to increases in telomere length that can result in chromosomal instability. These changes can increase replicative capacity and led to cell phenotypes similar to cancer cell lines. The ablation of the telomerase gene occurs through direct action of the Cas protein or by activating genes that inhibit its transcription (E). Overall, these changes lead to a decrease in replicative capacity and/or an upregulation in alternate telomere lengthening mechanisms. Imaging telomerase involves directly introducing a fluorescent tag onto the TERT protein (F). This allows for the study of telomerase activity as well as the dynamics of telomere formation.

Aging is a pervasive and complicated process that results from the body’s limited capacity to regenerate itself. Cancer is an almost as pervasive and equally complicated disease that hijacks these regenerative capabilities to proliferate unchecked. Due to their complex nature, parsing out the mechanisms of each appears to be a daunting task. However, the inception of CRISPR-Cas technology has provided a powerful tool that can be used to fashion rapid and specific genomic changes in living organisms. This system, as overviewed in Figure 3, has already made major contributions to the understanding of telomeres and telomerase in the context of aging and disease and it will undoubtedly continue to do so as the technology develops.
